# Comparative Structural Characterisation and In Vitro Bioactivities of Crude and Purified Polysaccharides from Dandelion (*Taraxacum officinale*) Root Using Microwave-Assisted Enzymatic Extraction

**DOI:** 10.3390/molecules31172966

**Published:** 2026-08-25

**Authors:** Jingwen Zhang, Fujuan Zhang, Yuxuan Liu, Cuntang Wang, Guojun Du

**Affiliations:** 1 College of Food and Bioengineering, Qiqihar University, Qiqihar 161006, Chinawangcuntang1980@163.com (C.W.); 2 Department of Biochemical Engineering, Chaoyang Normal University, Chaoyang 122000, China

**Keywords:** microwave-assisted compound enzymatic extraction, Dandelion root polysaccharides, structural characterisation, antioxidant activity, hypoglycaemic activity

## Abstract

The increasing demand for functional products has promoted research on bioactive compounds. Dandelion root polysaccharide (DRP), a key bioactive component of Taraxacum officinale, has anti-inflammatory, antioxidant, hypoglycemic and hypolipidemic activities. This study extracted DRP via microwave-assisted enzymatic extraction (MAEE) to obtain mDRP, and isolated acidic polysaccharide mDRP-1 (7.548 kDa) by column chromatography. HPLC showed that both contain six monosaccharides (Man, Rha, GalA, Glu, Gal, Ara) with different molar ratios. mDRP is heterogeneous (trace protein/nucleic acids, no triple helix, amorphous), while mDRP-1 has a triple-helical, semi-crystalline structure; both have characteristic polysaccharide peaks and pyranose rings, and rough, porous surfaces. mDRP had better thermal stability and significant concentration-dependent in vitro antioxidant and hypoglycaemic effects.

## 1. Introduction

Diabetes mellitus (DM) is a chronic metabolic disorder caused by insulin dysfunction, with Type 2 diabetes accounting for 90% of cases globally. Its hallmark hyperglycaemia can lead to multisystem complications [1]. While conventional pharmacological treatments are associated with adverse effects, natural compounds such as inulin and acarbose have been utilised in traditional medicine worldwide for managing hepatitis, cardiovascular diseases, and diabetes through blood glucose modulation and gut microbiota improvement [2]. Dandelion (*Taraxacum officinale*), a perennial herb of the Asteraceae family, is rich in bioactive constituents including polysaccharides, flavonoids, triterpenes, phenolic acids, and sesquiterpene lactones [3]. It exhibits diverse pharmacological properties, such as anticancer, anti-inflammatory, antioxidant, antimicrobial, and hypoglycaemic/hypolipidaemic effects [4,5,6,7,8]. Studies indicate that dandelion extracts containing polysaccharides and flavonoids, when combined with astragalus extract (RAE), demonstrate synergistic hypoglycaemic activity by significantly inhibiting α-glucosidase and α-amylase, ameliorating insulin resistance in IR-HepG2 cells, and enhancing hexokinase, pyruvate kinase activity, and glycogen synthesis [9]. Preliminary research also suggests that dandelion polysaccharides improve growth performance, antioxidant capacity, and immunity in carp [10]. Dandelion polysaccharides (e.g., DLP-I), extracted via ultrasound-assisted enzymatic extraction (UAEE), exhibit increased yield alongside immunomodulatory, hepatoprotective, and digestive stability properties [11]. Furthermore, chlorogenic acid derivatives, hydroxy phenylacetic acid inositol esters (PIEs), and amino acid-conjugated sesquiterpene lactones from dandelion roots demonstrate antioxidant, anticoagulant, and antiplatelet activation effects, suggesting therapeutic potential for cardiovascular diseases [4,12]. Incorporating dandelion root powder (≤3% *w*/*w*) into wheat bread enhances polyphenol content and antioxidant activity, though excessive amounts impair palatability [13]. Beyond its medicinal applications, dandelion holds promise in the food industry (e.g., inulin production), animal husbandry (e.g., feed additive), and global traditional medicine (e.g., hepatitis and diabetes management). However, challenges such as standardised production and elucidating bioactive mechanisms remain, necessitating interdisciplinary research to advance pharmacological and clinical translation [14].

In contemporary mainstream polysaccharide extraction technologies, enzyme-assisted extraction (EAE) achieves selective isolation through gentle degradation of cell wall structures, offering controlled operational parameters and environmental sustainability [15,16,17]. Representative applications include papain-mediated extraction of Sargassum polysaccharides [18] and enzymatic separation of pectin and inulin from artichoke [19]. Enzyme modification technology effectively enhances bioactivity through macromolecular depolymerisation and spatial conformation alteration [15]. Microwave-assisted extraction (MAE) employs electromagnetic waves for rapid cell wall disruption, combining extraction efficiency with bioactive preservation. Berkani et al. optimised MAE parameters for date palm polysaccharides, achieving a 13.98% experimental yield. The extracted polysaccharides demonstrated potent antioxidant activity and significant anti-inflammatory effects through inhibition of heat-induced and hypotonic hemolysis, along with stabilisation of erythrocyte membrane BSA proteins [20]. Cui et al. revealed superior protective effects of microwave-processed samples against LPS-induced oxidative damage in zebrafish embryo models compared to controls, evidenced by enhanced SOD, GSH-Px, and CAT activities coupled with reduced MDA levels [21]. Given persistent limitations in extraction efficiency imposed by plant cell walls, synergistic technologies such as MAEE and UAEE have emerged. These hybrid approaches combine enzymatic hydrolysis with physical disruption methods to significantly improve polysaccharide yields. For instance, Zhu Yawei et al. [10] achieved 15.23% MLPs extraction efficiency through enzyme-microwave coupling optimised via Box–Behnken design. In microalgal polysaccharide extraction, Peng et al. [22] developed MAEE technology integrating microwave-induced cell wall rupture with α-amylase digestion, simultaneously enhancing polysaccharide solubility and DPPH radical scavenging capacity through enzymatic modification [22]. Collectively, advancements in process optimisation (response surface methodology) and technological innovation (synergistic extraction systems, enzymatic modification) have substantially improved both extraction efficiency and the functional properties of polysaccharides, providing critical theoretical foundations for applications in the food and pharmaceutical industries.

Despite these advances, the application of MAEE to dandelion root polysaccharides (DRP) and the comparative evaluation of crude versus purified fractions remain unexplored. We previously established a microwave-assisted extraction (MAE) method for dandelion root polysaccharides (DRP) and optimised the conditions using response surface methodology [23]. That work achieved a yield of 24.85 ± 0.457% and provided preliminary characterisation of crude polysaccharides. However, two aspects could be further explored: the extraction efficiency might be improved by enzymatic pretreatment, and, more importantly, the structure–activity relationship as well as the effect of purification on bioactivity remain to be elucidated. The present study overcomes these limitations by employing microwave-assisted enzymatic extraction (MAEE) to improve yield, and adopts a simplified purification strategy using macroporous resin and cellulose column chromatography to obtain a single purified fraction (mDRP-1). Beyond reassessing antioxidant activity, this work introduces, for the first time, a systematic evaluation of hypoglycemic activity. The monosaccharide composition and molecular weight distribution of mDRP and mDRP-1 were analysed by HPLC before and after purification, and comprehensive structural characterisation was performed using FT-IR, XRD, NMR, SEM, and TG-DSC. Comparison between the crude and purified fractions reveals that mDRP-1 exhibits lower bioactivity than mDRP, suggesting that the efficacy of dandelion root polysaccharides derives from synergistic interactions among multiple components rather than a single purified entity. This study addresses a knowledge gap in the application of MAEE for DRP extraction and expands potential avenues for its efficient utilisation.

## 2. Results and Discussion

### 2.1. Physicochemical Properties of Polysaccharides

**Figure 1:**
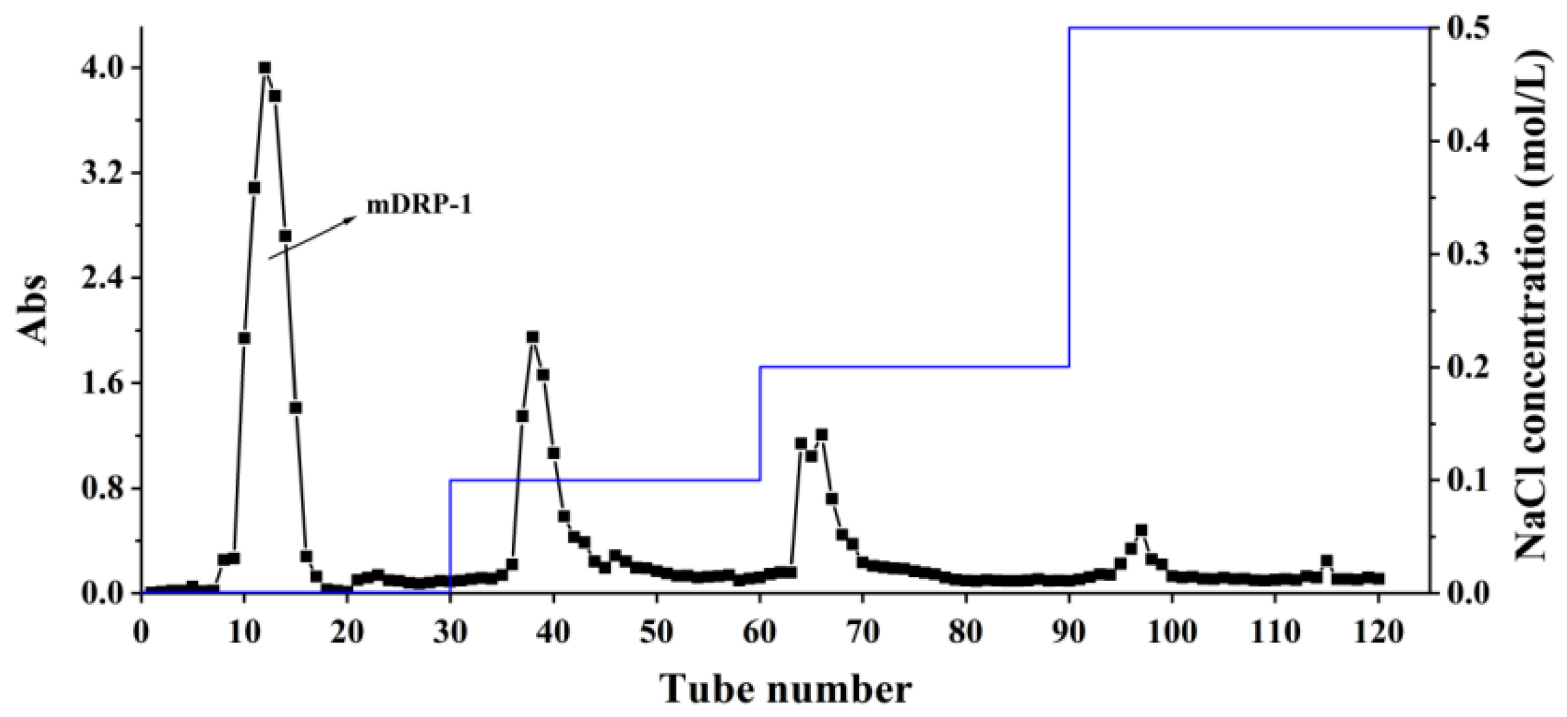
Elution diagram of mDRP by DEAE-52 column chromatography.

MAEE method was employed to extract polysaccharides from dandelion root, yielding mDRP. The mDRP was subsequently purified using macroporous resin chromatography followed by DEAE-52 Cellulose column chromatography. This process isolated the mDRP-1 fraction (Figure 1). Other fractions eluted with NaCl solution were not collected or retained for further analysis due to their low polysaccharide content, which rendered them unsuitable for detailed study. As presented in Table 1, the total sugar and protein contents of mDRP and mDRP-1 were determined. mDRP exhibited a total sugar content of 75.71% and a protein content of 1.63%. In contrast, the purified mDRP-1 fraction demonstrated a significantly higher total sugar content (85.35%) and a markedly lower protein content (0.36%). These results indicate effective polysaccharide purification, as the refined fraction displayed both enhanced carbohydrate purity and reduced protein contamination.

#### 2.1.1. Molecular Weight

**Figure 2:**
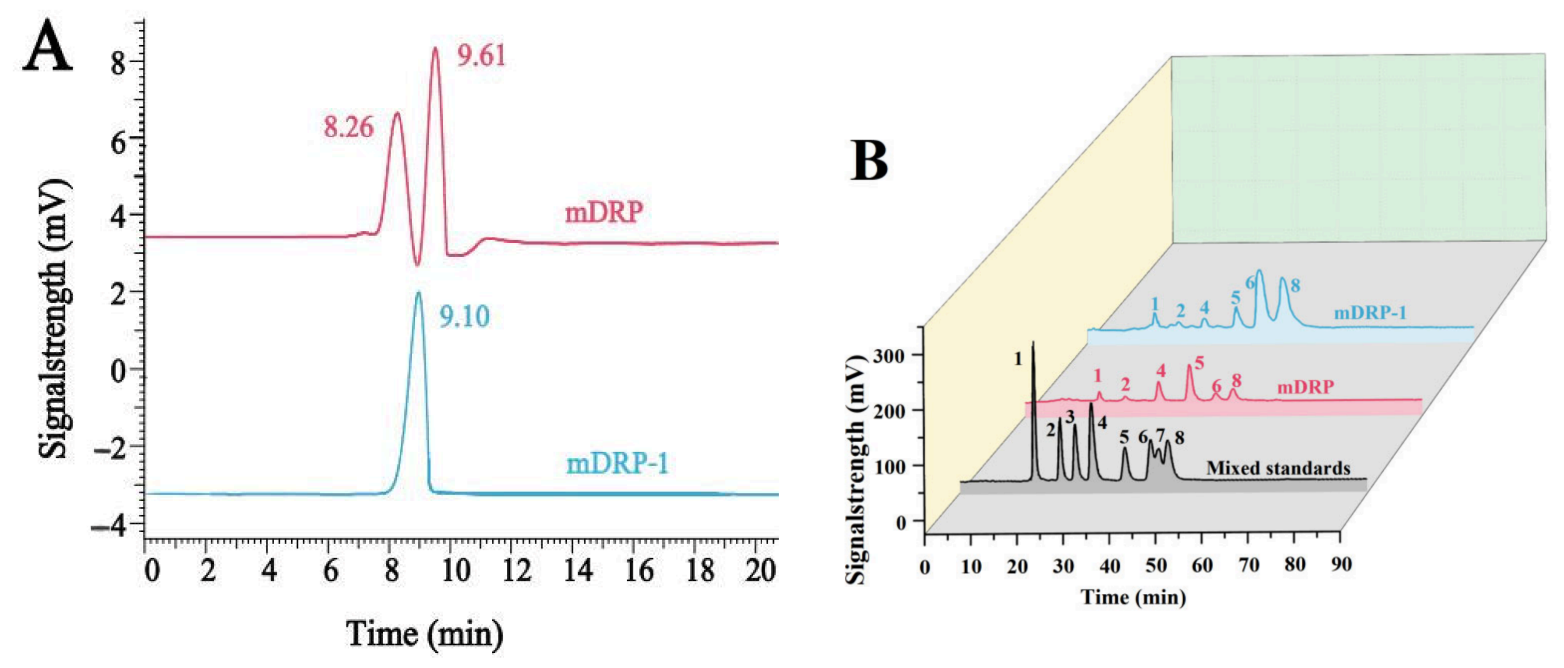
(**A**) HPGPC elution profile of mDRP and mDRP-1; (**B**) HPGPC profile of monosaccharide standard, mDRP and mDRP-1. Peaks: 1 Man, 2 Rha, 3 GlcA, 4 GalA, 5 Glu, 6 Gal, 7 Xyl, 8 Ara.

The bioactivity of polysaccharides is often correlated with their molecular weight [16]. HPGPC was used to determine the molecular weights of mDRP and mDRP-1 (Figure 2A, Table 1). The chromatogram of mDRP exhibited two symmetrical peaks, indicating that it is heterogeneous. In contrast, mDRP-1 displayed a single symmetrical peak, confirming its homogeneity, uniform molecular weight distribution, and high purity. Since mDRP showed two major peaks in the chromatogram, the average retention time method was adopted, and the average of the two retention times (8.935 min) was calculated and substituted into the calibration curve equation to estimate the molecular weight. Then, the average molecular weight of mDRP was calculated as 11.811 kDa, and the molecular weight of mDRP-1 was 7.548 kDa.

#### 2.1.2. Monosaccharide Composition

The monosaccharide composition serves as a fundamental unit of polysaccharides, and the varying combinations of these parameters hold significant value in polysaccharide research [16]. Analysis of HPLC results, by comparison with retention times of standard references (Figure 2), revealed that both mDRP and mDRP-1 were composed of Man, Rha, GalA, Glu, Gal, and Ara. The molar ratios of these monosaccharides were determined to be 0.43:0.27:2.58:7.6:1:1.82 (mDRP) and 0.35:0.21:0.31:1:6.03:4.75 (mDRP-1), respectively. Notably, glucose was the most abundant monosaccharide in mDRP, whereas mDRP-1 exhibited higher levels of arabinose and galactose, along with a relatively lower mannose content.

### 2.2. UV and FTIR

**Figure 3:**
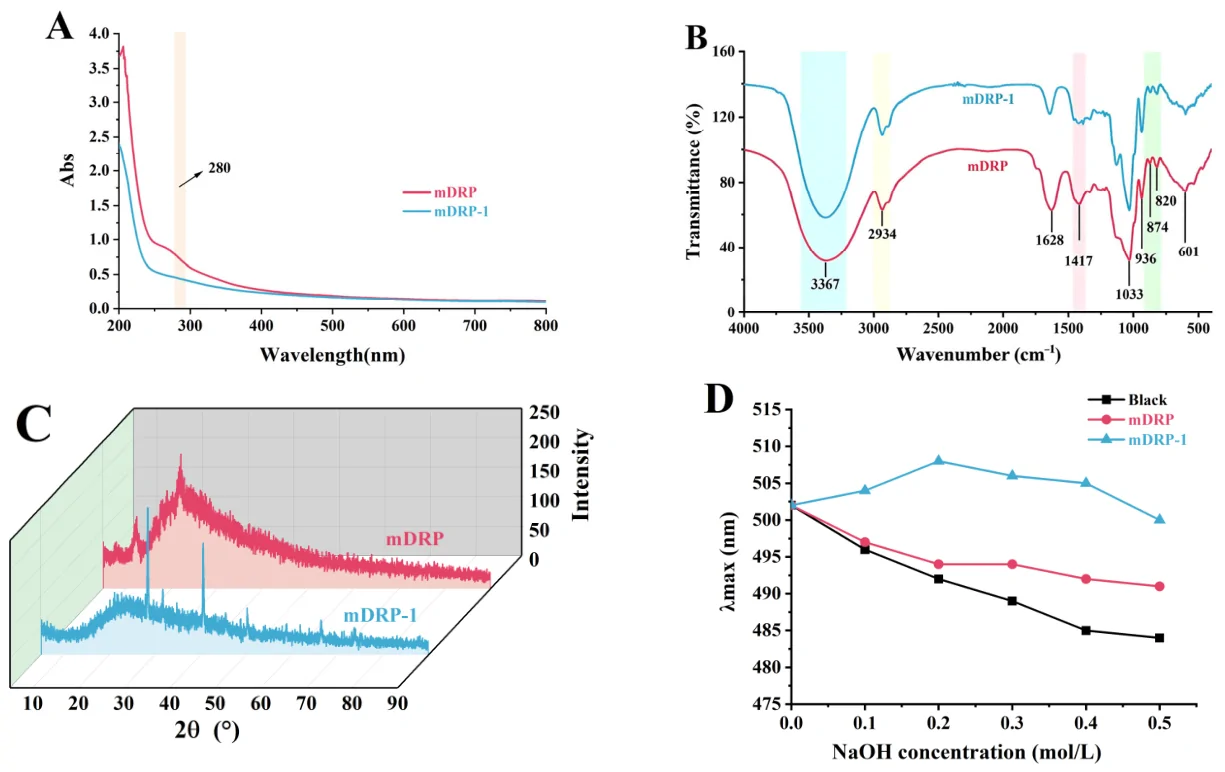
(**A**) UV spectra of mDRP and mDRP-1; (**B**) FT-IR spectra of mDRP and mDRP-1; (**C**) XRD spectra; (**D**) Congo red experimental data.

Ultraviolet-visible (UV-Vis) spectroscopy can be employed to investigate conformational changes in proteins during protein-ligand interactions. It is generally believed that the UV absorption peaks of nucleic acids and proteins are located at 260 nm and 280 nm, respectively [24]. FTIR spectroscopy serves as a particularly valuable technique for identifying characteristic organic groups in polysaccharides, with three distinctive spectral regions: the O-H stretching band (3200–3600 cm^−1^), C-H stretching band (2800–3000 cm^−1^), and the fingerprint region (700–1800 cm^−1^) [20,25]. As illustrated in Figure 3A, mDRP demonstrates pronounced absorption peaks between 260 and 280 nm, indicating the presence of proteins and nucleic acids. In contrast, mDRP-1 shows no detectable absorption at these wavelengths, confirming effective purification with negligible protein/nucleic acid content. Figure 3B reveals similar FTIR spectra for both mDRP and mDRP-1, exhibiting characteristic polysaccharide absorption bands. Both samples display a broad, intense peak at approximately 3367 cm^−1^ attributable to O-H stretching vibrations, confirming hydroxyl group presence [26]. The weak absorption at 2934 cm^−1^ arises from C-H stretching vibrations of aliphatic groups [26]. Peaks at 1628 cm^−1^ and 1417 cm^−1^ correspond to carbonyl (C=O) and carboxyl (-COOH) groups, respectively [24], suggesting the possible presence of uronic acid derivatives. The absorption at 1033 cm^−1^ likely results from C-O stretching vibrations in pyranose rings. Both polysaccharides exhibit characteristic absorption between 1000 and 1200 cm^−1^, attributable to C-O-C and C-O stretching vibrations of glycosidic linkages and hydroxylated carbons, confirming the presence of pyranose rings. Diagnostic signals at 820 cm^−1^ and 874 cm^−1^ indicate the coexistence of both α- and β-glycosidic linkages in their backbone structures [27]. Collectively, these spectroscopic data demonstrate that mDRP and mDRP-1 are typical acidic polysaccharides containing pyranose rings with both α- and β-glycosidic linkages.

### 2.3. XRD

X-ray diffraction (XRD) is a pivotal technique for characterising the crystalline structure of polysaccharides. By analysing diffraction peak profiles, it enables the differentiation between crystalline and amorphous phases. Crystalline materials exhibit sharp, narrow diffraction peaks, whereas amorphous components produce broad peaks [28]. Beyond elucidating molecular structure, XRD also facilitates the prediction of physicochemical properties such as swelling capacity, density, and solubility, thereby serving as a vital analytical tool for understanding structure-function relationships in polysaccharides [29,30]. As illustrated in Figure 3C, the XRD pattern of mDRP displays a weak, diffuse peak at 12° and a broad, hump-like peak near 22°, with no sharp reflections—indicating low crystallinity, a hallmark of typical polymeric materials [31]. In contrast, mDRP-1 exhibits a hump-shaped peak at 24° alongside sharp peaks at 28° and 40°, confirming its higher crystalline nature. These findings align with prior reports by Chen et al. [32]. The extraction and purification processes may partially disrupt polysaccharide crystallinity. The experimental results demonstrate that mDRP is predominantly amorphous, whereas mDRP-1 adopts a semi-crystalline state. The transition from amorphous to crystalline phases in polysaccharides is closely associated with their high molecular weight and structural complexity [33]. Crystalline architecture is primarily governed by structural parameters such as molecular weight and glycosidic linkage patterns [30]. These molecular-level structural features not only dictate crystallisation behaviour but also critically influence key physicochemical properties, including thermal stability and solubility [34].

### 2.4. Conformational Analysis

Congo Red, an acidic dye, can be used to detect the conformational characteristics of polysaccharide chains in aqueous solutions through specific binding assays. When a polysaccharide adopts a triple-helix structure, Congo Red can form a complex with its internal helical framework, inducing a red shift in the maximum absorption wavelength (λ_max_) of the solution [16]. This conformational transition primarily relies on the stabilising effect of hydrogen-bond networks on the helical structure [25]. The maximum wavelengths of mDRP and mDRP-1 in varying NaOH solutions are illustrated in Figure 3D. Notably, mDRP exhibited no red shift at any NaOH concentration, whereas the maximum absorption wavelength of mDRP-1 increased with rising NaOH concentration, confirming formation of a Congo Red-polysaccharide complex. These findings suggest that mDRP lacks a triple-helical conformation under the tested conditions and may instead possess a rigid structure resistant to highly alkaline conditions [25], consistent with the observations of Abu et al. [29]. In contrast, mDRP-1 appears to adopt a triple-helix-like conformation, which aligns with previous reports on purified polysaccharides DPR and DPL by Qiao et al. [11]. According to Guo et al. [35], formation mechanisms and analytical methods for triple-helical polysaccharides remain insufficiently explored. Polysaccharides devoid of triple-helix structures typically exhibit lower structural order than their triple-helical counterparts.

### 2.5. NMR

**Figure 4:**
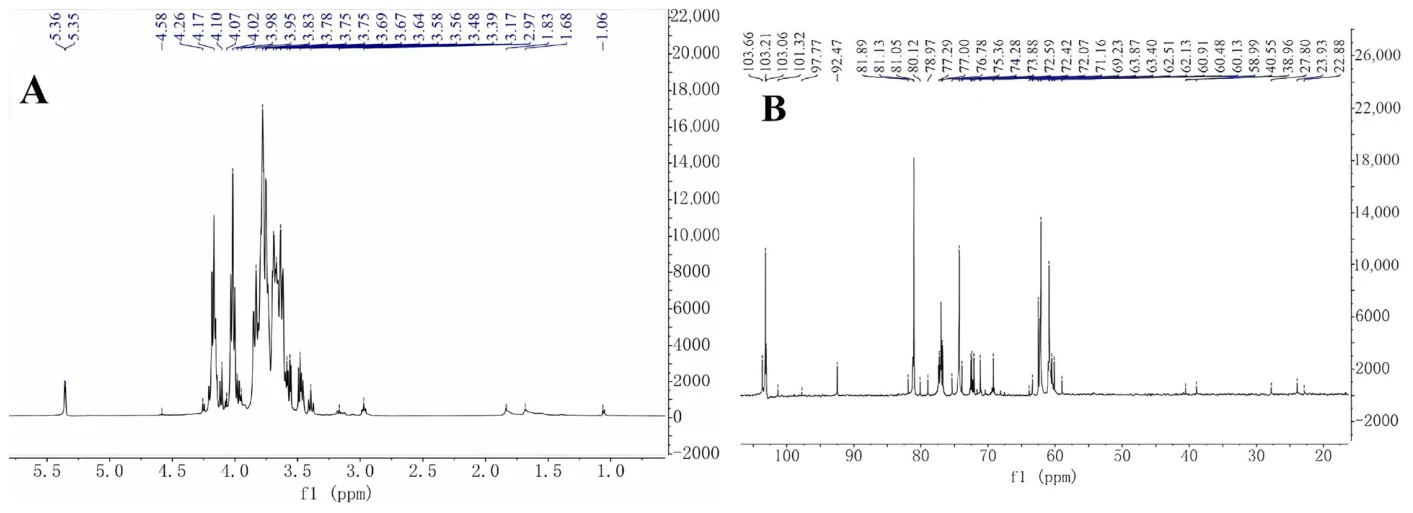
NMR spectra of mDRP-1: (**A**) ^1^H NMR spectrum; (**B**) ^13^C NMR spectrum.

NMR is a pivotal technique for elucidating the structure of polysaccharides, providing atomic-level resolution and indispensable structural information, including the sequence of monosaccharide residues, anomeric carbon configurations, and glycosidic linkage patterns [36,37]. The NMR analysis of mDRP-1, including its^1^H and ^13^C spectra, is presented in Figure 4.

Typically, signals above 4.95 ppm correspond to α-configured sugar residues, whereas those below 4.95 ppm indicate β-configured residues [32]. In the ^1^H NMR spectrum of mDRP-1 (Figure 4A), signals were predominantly distributed within the δ 3.2–6.0 ppm range. Notably, anomeric proton signals at δ 4.5–5.0 ppm exhibited characteristic β-configuration peaks, suggesting the presence of β-glycosidic linkages in the backbone [32]. In contrast, α-configured anomeric protons typically resonate at δ 5.0–6.0 ppm, and a distinct α-configuration signal was observed near δ 5.36. Concurrently, the ^13^C NMR spectrum (Figure 4B) displayed anomeric carbon signals at δ 103.66 and δ 103.21 (β-configuration) as well as δ 92.47 (α-configuration), which are consistent with the coexistence of both α- and β-configurations in mDRP-1, consistent with FTIR spectroscopic findings. Furthermore, the non-anomeric carbon signals (δ 60.1–81.0 ppm) in mDRP-1′s ^13^C spectrum exhibited characteristic pyranosyl ring features [36]. Signals observed at δ 77.29–81.89 may be associated with 1→3 and 1→4 glycosidic linkages, while signals corresponding to C6 hydroxyl substitutions (δ 62.13–63.40) and distinctive peaks at δ 27.80 and δ 23.93 further supported a branched structure, potentially including deoxy sugar modifications. Collectively, these findings provide preliminary evidence that the highly branched nature of mDRP-1 may contribute to its notable water retention and thickening properties [38]. The polysaccharide comprises both α- and β-configurations, featuring pyranosyl ring signatures and deoxy sugar modifications, corroborating the FTIR data. However, it should be emphasised that detailed structural characterisation of mDRP-1 necessitates further investigation via periodate oxidation and methylation analysis. Additionally, the pyranosyl backbone signals (δ 60–85 ppm) and the multiplet distribution in the anomeric region require validation through two-dimensional NMR techniques (HSQC and COSY) to ascertain the sequence of glycosyl units and the nature of substituents. Such studies are essential to elucidate structure-function relationships and assess the potential applications of mDRP-1 in food science.

### 2.6. SEM

**Figure 5:**
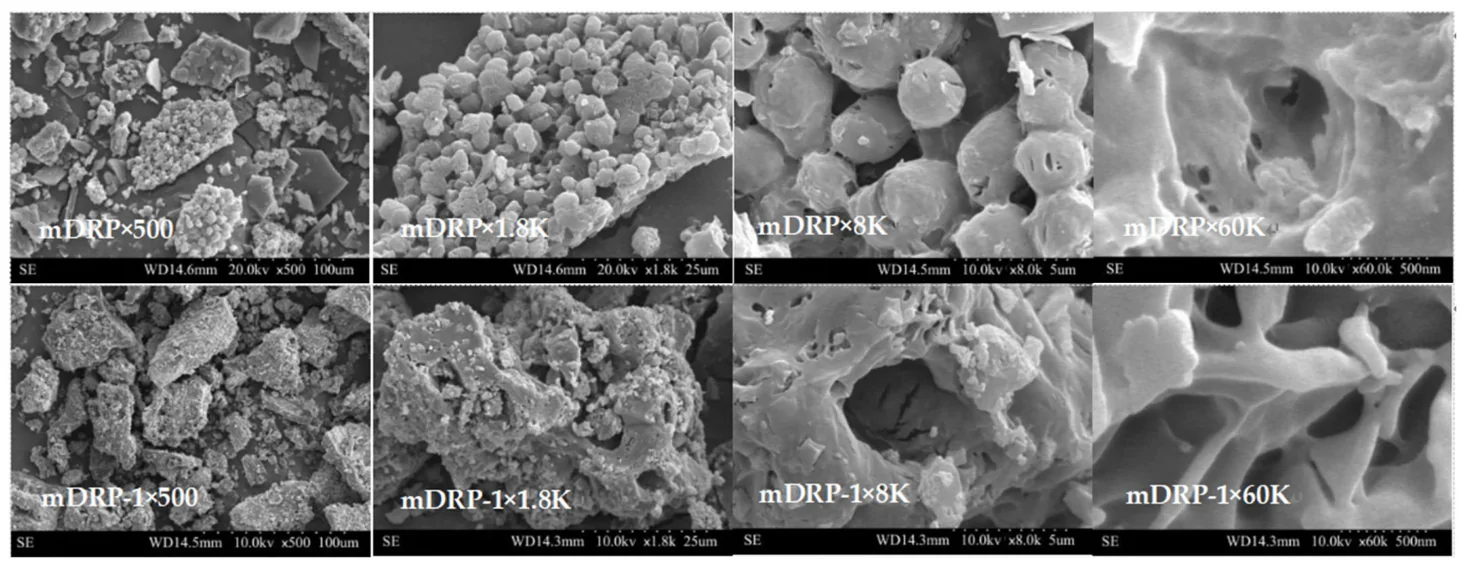
The morphological characterisation of mDRP and mDRP-1 obtained by different extraction methods at 500×, 1.8K×, 8K× and 60K× magnifications.

The biological functions of polysaccharides are closely associated with their three-dimensional conformation, as their complex structural configurations significantly influence bioactive expression. Different extraction methodologies can markedly alter the aggregation state and surface morphology of polysaccharides. SEM, owing to its high resolution, extensive depth of field, and superior surface topography characterisation, has emerged as the most effective technique for elucidating the microstructure of polysaccharides, allowing direct visualisation of intricate stereochemical details [30]. As illustrated in Figure 5, mDRP and mDRP-1 exhibit certain morphological similarities. Specifically, mDRP presents as flaky or granular clusters with a rough surface containing unevenly distributed fissures and pores—a structural resemblance to the *Rubus crataegifolius* root polysaccharides (RCP-3) reported by Chen et al. [32]. This phenomenon may be attributed to variations in monosaccharide composition under microwave irradiation and the influence of ethanol on the surface architecture of mDRP [24]. Lin et al. [39] and Guo et al. [40] postulated that MAE induces intracellular water vaporisation via microwave dielectric heating, generating steam pressure that causes cell wall shrinkage, rupture, and crack formation. In contrast, EAE directly degrades cell wall components through enzymatic hydrolysis. The synergistic application of these techniques substantially exacerbates the disruption of cell walls and membranes, resulting in extensive voids and cavities. The SEM micrograph of mDRP-1 reveals aggregated masses with a coarse surface and larger pores, resembling the structure of microwave-extracted dandelion root polysaccharides obtained in our prior study. Collectively, SEM analysis demonstrates that both polysaccharides exhibit dense, irregular surfaces with concavities, and the combined use of MAE and EAE facilitates enhanced polysaccharide release.

### 2.7. TG-DSC

Thermal analysis plays a pivotal role in food processing and polysaccharide research [24]. Thermogravimetry (TG), which monitors mass changes induced by dehydration, decomposition, and oxidation, serves as an effective method for evaluating the thermal stability of polysaccharides [30]. Its derivative thermogravimetric (DTG) curve precisely reflects the rate of mass loss and peak temperatures. Meanwhile, differential scanning calorimetry (DSC) analyses structural deformation, crystalline melting, and intermolecular interactions by recording heat flow-temperature differentials, with endothermic/exothermic peaks revealing physicochemical transitions. The thermodynamic properties of polysaccharides are closely linked to their structure and functional groups, and these thermal analysis techniques collectively provide a systematic framework for investigating material stability, decomposition behaviour, and potential biological applications [25].

**Figure 6:**
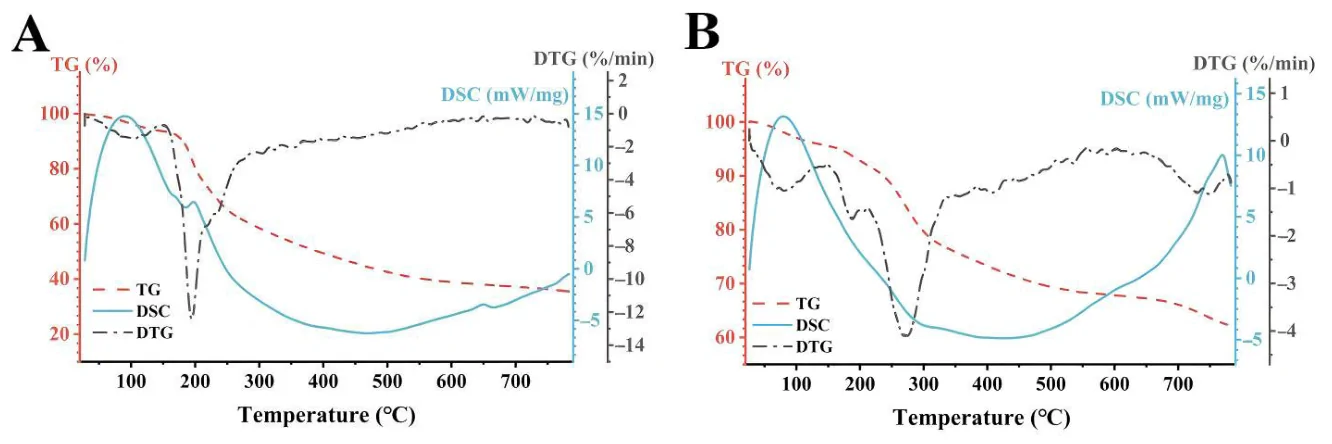
Thermal properties of mDRP (**A**) and mDRP-1 (**B**).

As shown in Figure 6, TG curves revealed a three-stage degradation pattern for both samples. The first minor mass loss (20–170 °C) was attributed to evaporation of free and bound water in the polysaccharides [30]. A second, more substantial mass loss occurred at 240–650 °C for mDRP and 200–630 °C for mDRP-1, characterised by rapid weight reduction due to intense decomposition reactions involving cleavage of glycosidic bonds, carbon chains, and hydrogen bonds [24]. The third stage (650–783 °C for mDRP-1; 630–783 °C for mDRP) exhibited slower mass loss, associated with further degradation of depolymerised polysaccharides [41]. At 783 °C, the residual masses were 60.09% for mDRP-1 and 35.52% for mDRP. The DTG curves indicated maximum mass loss rates at 271.84 °C and 195.43 °C, respectively, with significantly slower degradation thereafter. DSC analysis revealed one exothermic peak (90.89 °C) and one endothermic peak (469.24 °C) for mDRP-1, whereas mDRP displayed two exothermic peaks (82.48 °C, 771.54 °C) and one endothermic peak (398.04 °C). These results demonstrate that mDRP exhibits higher thermal stability than mDRP-1, as evidenced by its higher decomposition temperatures and greater residual mass at 783 °C. The observed differences in thermal behaviour may be related to the distinct structural features of the two polysaccharides, including differences in molecular conformation, monosaccharide composition, and molecular weight. However, the specific structural determinants responsible for the enhanced thermal stability of mDRP cannot be conclusively identified from the current data and require further investigation through systematic structural modification and correlation analysis.

### 2.8. In Vitro Antioxidant Activities

**Figure 7:**
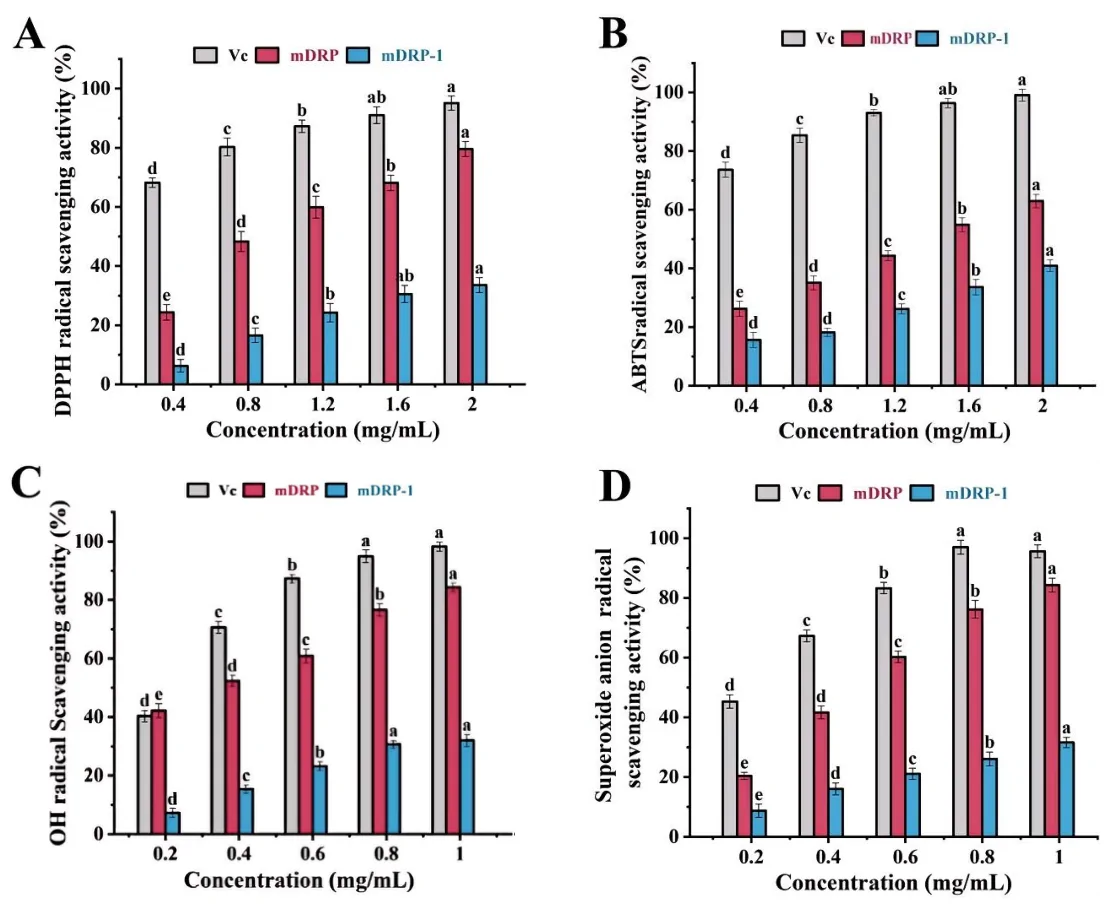
Antioxidant activity of polysaccharides in vitro: (**A**) Scavenging activity on DPPH free radical; (**B**) Scavenging activity on ABTS free radical; (**C**) Scavenging activity on hydroxyl free radical; (**D**) Scavenging activity on Superoxide anion. Different lowercase letters (a–e) above bars indicate significant differences among groups as determined by Duncan’s multiple range test (*p* < 0.05).

Polysaccharides mitigate oxidative damage through multiple pathways to scavenge reactive oxygen species (ROS), and their antioxidant effects have been validated in both in vivo and in vitro models. The DPPH radical scavenging assay evaluates the hydrogen-donating capacity of polysaccharides, reflecting the activity of phenolic hydroxyl groups and other functional groups in hydrophobic environments. The ABTS assay focuses on the electron transfer ability of water-soluble polysaccharides in hydrophilic media. The hydroxyl radical scavenging experiment reveals the mechanism by which polysaccharides protect biomacromolecules through hydrogen atom transfer. Meanwhile, the superoxide anion scavenging assay assesses the efficacy against ROS precursors. The distinct chemical properties of different radicals lead to variations in the mechanisms of polysaccharide action. Integrating multiple models enables systematic analysis of the antioxidant characteristics of polysaccharides in both hydrophobic and hydrophilic environments, providing a theoretical basis for delaying food oxidation and preventing/treating oxidative stress-related diseases [16,30]. As shown in Figure 7, the scavenging effects of mDRP and mDRP-1 on free radicals and superoxide anions gradually increased with concentration, though their efficacy remained lower than that of vitamin C (Vc). At 2.0 mg/mL, both mDRP and mDRP-1 exhibited optimal DPPH and ABTS radical scavenging activities, consistent with findings reported by Cai et al. [42]. At 1.0 mg/mL, these polysaccharides demonstrated peak hydroxyl radical and superoxide anion scavenging capacities. Quantitative analysis using half-maximal inhibitory concentration (IC_50_) revealed that in the DPPH radical scavenging system, the IC_50_ values for mDRP and mDRP-1were 0.87 mg/mL and 3.27 mg/mL, respectively. For ABTS radical scavenging, the corresponding IC_50_ values were 1.32 mg/mL and 3.48 mg/mL, aligning with Feng et al. [43] results on the antioxidant properties of lotus leaf polysaccharides. The IC_50_ values for hydroxyl radical scavenging were 0.31 mg/mL and 1.77 mg/mL, respectively. In the superoxide anion scavenging system, mDRP and mDRP-1 showed IC_50_ values of 0.45 mg/mL and 2.29 mg/mL, respectively. However, these data represent in vitro chemical reactivity rather than in vivo efficacy, and the observed activity differences between samples should be interpreted as correlative observations rather than mechanistic conclusions.

Studies evaluating antioxidant activity demonstrated that both mDRP and mDRP-1 exhibited characteristic concentration-dependent positive correlations in antioxidant assays. Among these, mDRP displayed optimal antioxidant activity, albeit significantly inferior to Vc. Notably, whilst the purified mDRP-1 showed substantially reduced activity compared to mDRP, this phenomenon aligns with the pattern of post-purification activity alterations in polysaccharides previously reported by Li et al. [26]. The antioxidant capacity of polysaccharides typically results from synergistic multifactorial effects. Several factors may explain the reduced activity of mDRP-1, including the removal of synergistic bioactive constituents, purification-induced structural modifications, or loss of minor active components. However, the precise mechanism remains unclear without further reconstitution and mechanistic studies. In conclusion, the in vitro data suggest that mDRP has the potential to act as a multi-target radical scavenger, demonstrating promising applications in preventing oxidative stress damage through its diverse antioxidant mechanisms.

### 2.9. In Vitro Hypoglycemic Activities

**Figure 8:**
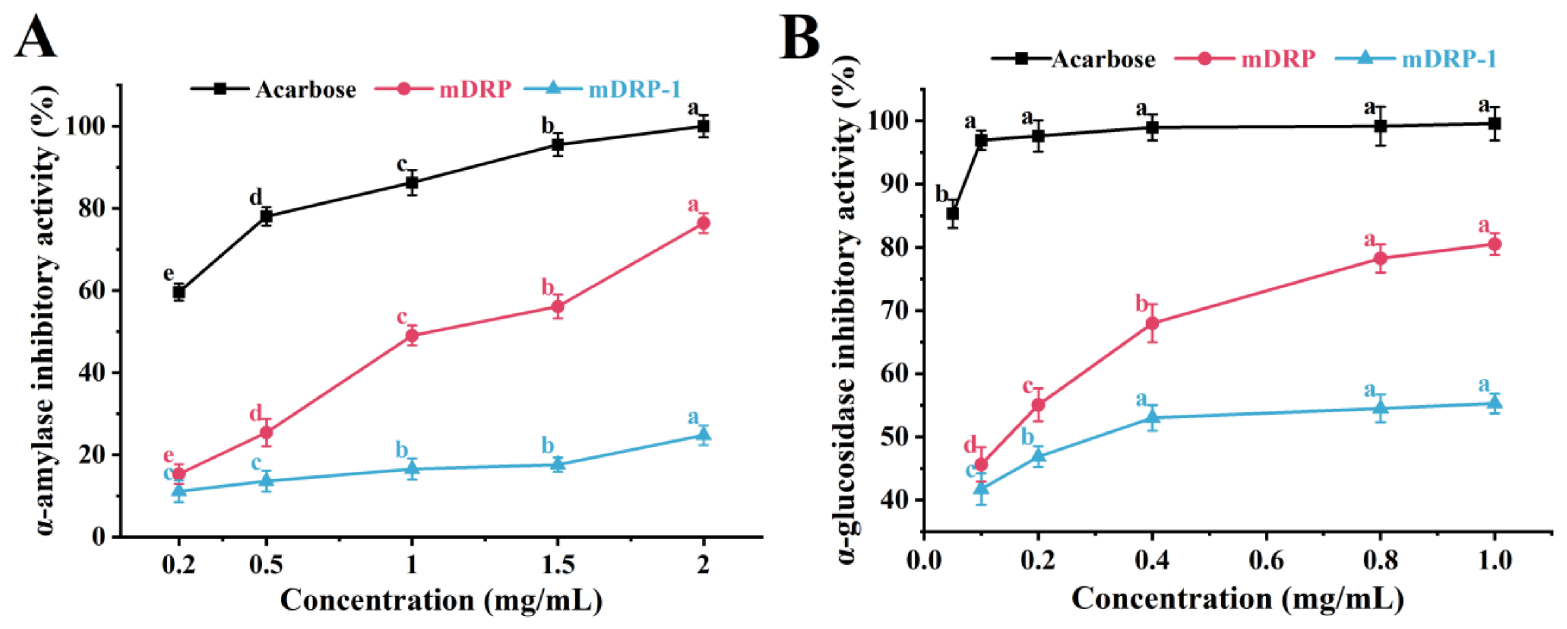
Hypoglycaemic activity of polysaccharides in vitro: (**A**) Inhibitory activity on α-glucosidase; (**B**) Inhibitory activity on α-amylase. Different lowercase letters (a–e) above bars indicate significant differences among groups as determined by Duncan’s multiple range test (*p* < 0.05).

As pivotal enzymes in dietary carbohydrate digestion, α-amylase and α-glucosidase represent key targets for delaying sugar absorption through enzymatic inhibition. This in vitro model offers distinct advantages, including robust controllability, cost-effectiveness, and operational safety. Research demonstrates that polysaccharides exhibit glycaemic-regulatory potential via specific inhibition of these digestive enzymes, thereby establishing a scientific foundation for developing natural polysaccharide-based functional foods for glycaemic control [28]. As illustrated in Figure 8A, mDRP exhibited a higher inhibitory rate against α-glucosidase compared to the purified fraction mDRP-1. This enhanced activity may be associated with the synergistic effects of other constituents present in mDRP beyond polysaccharides. However, all tested samples demonstrated significantly lower inhibitory rates than acarbose. Within the 0.1–1 mg/mL concentration range, mDRP displayed dose-dependent α-glucosidase inhibition, whereas mDRP-1 exhibited negligible concentration-response effects. At 2.0 mg/mL, mDRP inhibited 80.52% of α-glucosidase activity versus merely 55.28% for mDRP-1, with IC_50_ values determined as 0.136 mg/mL and 0.358 mg/mL, respectively. Figure 8B illustrates that between 0.2 and 2 mg/mL, mDRP demonstrated concentration-responsive α-amylase inhibition, contrasting with mDRP-1’s minimal response. Both polysaccharides showed inferior inhibitory effects to acarbose across all tested concentrations. At 1.0 mg/mL, mDRP achieved 76.41% α-amylase inhibition compared to mDRP-1’s 24.76%, with an IC_50_ of 1.005 mg/mL for mDRP.

Research indicates that the differential inhibitory activity of mDRP against α-amylase and α-glucosidase is intrinsically linked to their structural characteristics, including monosaccharide composition, molecular weight, surface morphology, and protein content [31,43]. Microwave treatment reduces polysaccharide molecular weight while enhancing binding capacity to enzymes through hydrogen bonding and hydrophobic interactions, which may contribute to mDRP’s superior inhibitory activity [31]. Concurrently, elevated protein content may potentiate its α-glucosidase inhibition [43,44]. Although Abu et al. [29] and Chen et al. [45] observed that lower molecular weight, coupled with higher arabinose and xylose levels, correlated with stronger inhibition, the present study demonstrates different findings, underscoring the complexity of structure-function relationships. In summary, mDRP exhibits notable antioxidant and hypoglycaemic properties, offering dual protective effects against oxidative stress and glycaemic dysregulation. While mDRP-1 displays fundamental hypoglycaemic capacity, mDRP demonstrates potential as a natural α-glucosidase inhibitor with favourable contrast to clinical agents like acarbose—which carries adverse effects including abdominal distension [46]. The in vitro bioactivity results, while promising, are preliminary in nature and require further validation through cell-based and in vivo studies.

## 3. Materials and Method

### 3.1. Materials and Reagents

Dandelion roots were sourced from Changbai Mountain, Jilin Province, China. Dextran standards, standard monosaccharides and bovine serum albumin were obtained from Beijing Feimo Biotechnology Co., Ltd. (Beijing, China). Macroporous resin was purchased from Hebei Kaidi New Materials Company (Cangzhou, China), and DEAE-52 cellulose was sourced from Phygene Biotechnology Co., Ltd. (Fuzhou, China). All other chemicals and solvents used in this study were of analytical grade.

### 3.2. Extraction of mDRP

Polysaccharides from dandelion roots were extracted by the MAEE method [22]. First, the cleaned dandelion roots were dried in an oven at 70 °C, then ground into powder using a high-speed grinder. The dandelion root powder was then defatted using petroleum ether. Subsequently, the defatted powder was mixed with distilled water at a solid-to-liquid ratio of 1:20 (g/mL). Enzymatic hydrolysis was initiated by adding α-amylase (25 U/mL), cellulase (18 U/mL), and protease (280 U/mL; trypsin:papain = 2:1). The suspension was adjusted to pH 6 and transferred to a Sineo Microwave (MAS-II, Shanghai, China). Extraction was conducted at 300 W (intermittent irradiation mode), 50 °C for 30 min. Post-extraction, the mixture was inactivated by boiling, cooled to room temperature, and vacuum-filtered. Polysaccharides were precipitated by adding four volumes of absolute ethanol, followed by overnight storage at 4 °C. The precipitate was isolated by centrifugation (4000 rpm, 5 min), redissolved in distilled water, and lyophilised to yield crude polysaccharides (mDRP). Extraction yield was calculated using the Zhao et al. formula [47]. Protein content was quantified by the Bradford assay at 595 nm with bovine serum albumin as the standard [11]. The calibration curve (R^2^ = 0.9951) was: y=0.0074x+0.0202 Where y denoted absorbance and x denoted protein concentration (linear range: 0.02–0.1 mg/mL). Polysaccharide content was determined by the phenol-sulphuric acid method (PSAM) at 490 nm, with glucose as the standard [11]. The calibration curve (R^2^ = 0.9995) was defined as y=0.0111x+0.0024 Where y was absorbance, and x was polysaccharide concentration (linear range: 0.02–0.1 mg/mL).

### 3.3. Separation and Purification of mDRP

Following the methodology described by Wang et al. [1], S-8 macroporous resin was selected for decolourisation and deproteinisation of mDRP, based on the physicochemical properties of the polysaccharide. The S-8 resin was pre-treated by immersion in ethanol followed by acid-base activation, then wet-packed into a chromatography column (2.6 × 30 cm). The packed column was equilibrated overnight. The mDRP solution (3 mg/mL) was loaded onto the column, which was then eluted with distilled water at a flow rate of 2 BV/h. Fractions were collected at 5 min intervals, and polysaccharide content was quantified using the phenol-sulfuric acid method [48]. The major elution fractions were pooled and lyophilised to obtain decolourised and deproteinised dandelion root polysaccharide. For further purification, the crude polysaccharide was processed using DEAE-52 Cellulose chromatography (3.5 × 30 cm) according to Bai et al. [48]. After acid-base pre-treatment, the DEAE-52 Cellulose resin was wet-packed and equilibrated overnight. The decolourised and deproteinised polysaccharide solution (10 mg/mL, filtered through a 0.45 μm membrane) was loaded onto the column and eluted with a linear NaCl gradient (0–0.5 mol/L) at 1 mL/min. Eluates (5 mL/tube) were monitored at 490 nm via PSAM, with an elution profile plotted. The major peak fractions were combined, dialysed (3500 Da dialysate membrane, 48 h), and lyophilised to yield purified mDRP-1 for subsequent analysis. Polysaccharide and protein contents were determined before and after purification using PSAM and Coomassie Brilliant Blue Method (CBBM) [11], respectively.

### 3.4. Molecular Weight Determination

According to a previous study by Chen et al. [32], molecular weights (Mw) of mDRP and mDRP-1 were determined by high-performance gel permeation chromatography (HPGPC). A single symmetrical peak in the chromatogram indicated that the sample consisted of a homogeneous polysaccharide. A series of dextran standards (Dextran T5–T500) were sequentially injected, and their retention times (T) were recorded. The standard curve was drawn with T as the horizontal coordinate and LgMw as the vertical coordinate. The linear regression equation was: LgMw=−1.1646T+14.478 (R^2^ = 0.9986). For sample preparation, the polysaccharide fractions were accurately weighed and dissolved in ultrapure water to prepare 2 mg/mL solutions. The molecular weights of polysaccharides were calculated based on their T in the HPGPC analysis.

### 3.5. Determination of Monosaccharide Composition

Using the previously studied determination method, HPLC combined with a 1-phenyl-3-methyl-5-pyrrolidinone (PMP) column pre-determination was used to detect the monosaccharide composition of mDRP and mDRP-1 [23]. Approximately 3.0 mg of mDRP and mDRP-1 were accurately weighed and hydrolysed with 1 mL of 4 mol/L trichloroacetic acid in sealed ampoules at 120 °C for 4 h. The hydrolysate was subsequently evaporated to dryness under reduced pressure with methanol (repeated three times). The residue was then reconstituted in 2 mL of 0.3 mol/L NaOH solution to obtain the hydrolysed samples. For calibration, a standard solution was prepared by dissolving eight monosaccharide reference standards in appropriate concentrations. Aliquots (0.5 mL) of both the hydrolysed samples (mDRP and mDRP-1) and the standard monosaccharide solution were separately derivatised with 0.5 mL of 0.5 mol/L PMP solution. The mixtures were incubated at 70 °C for 30 min, followed by neutralisation with 0.5 mL of 0.3 mol/L HCl after cooling. The derivatised products were then extracted three times with 3 mL of chloroform, and the aqueous layer was filtered through a 0.22 μm membrane prior to HPLC analysis. Chromatographic separation was performed on a PRIMAIDE HPLC system (Hitachi, Japan) equipped with a DAD detector (λ = 250 nm). The injection volume was 20 μL, with the column maintained at 30 °C. The mobile phase consisted of 20 mmol/L phosphate buffer (pH 6.7) and acetonitrile (83:17, *v*/*v*) delivered at a flow rate of 1.0 mL/min. Monosaccharide identification and quantification were achieved by comparing retention times and peak areas of the derivatives with those of the standard monosaccharides. It should be noted that PMP derivatisation selectively targets reducing aldoses, whereas ketoses such as fructose are poorly derivatised under these conditions. Accordingly, the reported monosaccharide profiles predominantly reflect the aldose fraction of the polysaccharides.

### 3.6. UV Spectra Analysis

The 5 mg/mL polysaccharide solution was scanned using a UV (756PC, Qinghua Technology Co., Shanghai, China) across a range of 200–800 nm. The ultraviolet absorption spectra of mDRP and mDRP-1 were plotted, and the presence of absorption peaks at 260 mm and 280 mm was examined [31].

### 3.7. FTIR Spectra Analysis

The polysaccharide sample was mixed with KBr powder, and the blended mixture was ground and pressed into a thin pellet. The pellet was then placed in the detector and analysed using an FT-IR spectrometer (Nicolet, Thermo Fisher Scientific, Madison, WI, USA) with scanning performed over the range of 4000–400 cm^−1^ [31].

### 3.8. XRD Analysis

The crystalline structure of the polysaccharide was determined using X-ray diffraction (XRD) on a D8 Advance diffractometer (Bruker AXS, Karlsruhe, Germany). Following the method described by Mutaillifu et al. [49], XRD scans were carried out at room temperature with a scanning speed of 1°/min and a 2θ range of 5–80°. The generator was set at 40 kV and 40 mA.

### 3.9. Congo Red Experiment

The Congo red assay was performed with slight modifications based on the method described by Geng et al. [16]. Briefly, mDRP and mDRP-1 were prepared as solutions (2 mg/mL). The polysaccharide solutions were mixed with an equal volume of Congo red solution (0.2 mmol/L), followed by addition of NaOH solution (1 mol/L) to achieve final NaOH concentrations ranging from 0 to 0.5 mol/L. After reacting at room temperature for 5 min, distilled water was used as the blank control. The maximum absorption wavelength was recorded using a microplate reader (SpectraMax M3, Molecular Devices, San Jose, CA, USA), with spectral scanning performed at wavelengths ranging from 400 nm to 600 nm.

### 3.10. NMR Spectra Analysis

Following the methodology described by Chen et al. [27], dissolve 50.0 mg of sample in D_2_O, heat the mixture, and perform ultrasonication to complete deuterium exchange; subsequently, freeze-dry the mixture three times, followed by re-dissolution in 1.0 mL of D_2_O. Add 0.5 mL of the sample solution to an NMR tube, place the NMR tube into a nuclear magnetic resonance (NMR) spectrometer, and acquire one-dimensional ^1^H-NMR and ^13^C-NMR spectra of mDRP-1 at 25 °C. Data processing was performed using MestReNova 14 software.

### 3.11. SEM Determination

According to the method described by Yang et al. [24], a small quantity of the freeze-dried polysaccharide sample was mounted on conductive adhesive and subjected to gold sputtering coating. The sample was then observed and imaged using a scanning electron microscope (SEM; model S-3400, Hitachinaka, Ibaraki, Japan) at an accelerating voltage of 3 kV.

### 3.12. Antioxidant Activity In Vitro

The DPPH radical scavenging potential of dandelion root polysaccharides was evaluated using the analytical procedure established by Li et al. [26]. For ABTS radical scavenging assessment, an adapted experimental protocol derived from Yang et al. [24] was implemented. Hydroxyl radical neutralisation efficacy was quantified according to the standardised methodology reported in Li et al. [26]. Superoxide anion scavenging performance was analysed following the spectrophotometric approach developed by Yuan et al. [50]. Vitamin C (Vc) was used as the positive control in all antioxidant assays.

### 3.13. Determination of In Vitro Hypoglycaemic Activity

#### 3.13.1. Determination of α-Glucosidase Inhibitory Activity

Following the methodology of Ren et al. [46] with minor modifications, all solutions were initially prepared using 0.2 M phosphate-buffered saline (PBS) as the solvent. First, prepare all solutions using 0.2 M phosphate-buffered saline (PBS, pH 7.0). Next, mix the polysaccharide sample with α-glucosidase (5 U/mL) in equal volumes and react at room temperature for 10 min. Then add the p-nitrophenyl-α-D-glucoside solution (PNPG) and continue reacting for 20 min. Finally, add the Na_2_CO_3_ solution to terminate the reaction. Measure the absorbance at 400 nm using a microplate reader; use acarbose as the positive control.

$$\alpha \text{-}\mathrm{glucosidase}\;\mathrm{inhibitory}\;\mathrm{rate}\;(\%)=\left(1-\frac{A_{1}-A_{2}}{A_{0}}\right)\times 100\%$$

where A_1_ is the absorbance of samples with different concentrations; A_2_ is the absorbance of PBS instead of the α-glucosidase group; and A_0_ is the absorbance of PBS instead of the sample group.

#### 3.13.2. Determination of α-Amylase Inhibitory Activity

The α-amylase inhibition rate was assessed following the methodology of Abu et al. [29]. Briefly, prepare a 3 U/mL α-amylase solution and a 1% soluble starch solution using PBS. Add different concentrations of polysaccharide solutions to the α-amylase solution and incubate at 37 °C in a shaking incubator for 15 min. Then add the starch solution and react for 5 min. Finally, add DNS reagent to terminate the reaction. After a boiling water bath, measure the absorbance at 540 nm using a microplate reader; use acarbose as the positive control.

$$\alpha \text{-}\mathrm{amylase}\;\mathrm{inhibitory}\;\mathrm{rate}\;(\%)=\left(1-\frac{A_{1}-A_{2}}{A_{0}}\right)\times 100\%$$

where A_1_ is absorbance of samples with different concentrations; A_2_ is absorbance of PBS instead of α-amylase group; and A_0_ is absorbance of PBS instead of sample group.

### 3.14. Statistical Analysis

All assays were performed in triplicate (three independent experiments, each with three technical replicates). Data were expressed as mean ± standard deviation (SD). Statistical significance was assessed by one-way analysis of variance (ANOVA) followed by Duncan’s multiple range test, with a significance threshold of *p* < 0.05. Normality of data distribution was verified before performing ANOVA. All statistical analyses were performed using IBM SPSS Statistics 26.

## 4. Conclusions

Unlike our previous work, which focused on method development and preliminary characterisation, the present study employs MAEE, expands bioactivity evaluation to include hypoglycemic effects, and provides a direct comparative analysis of crude versus purified fractions. The successful application of MAEE for dandelion root polysaccharides demonstrates its potential as an efficient strategy for recovering bioactive polysaccharides from plant sources. The average molecular weights of mDRP and mDRP-1 were determined to be 11.811 kDa and 7.548 kDa, respectively. Based on PMP-HPLC analysis, both polysaccharides were found to contain Man, Rha, GalA, Glu, Gal, and Ara as the major detectable aldoses, with molar ratios of 0.43:0.27:2.58:7.6:1:1.82 for mDRP and 0.35:0.21:0.31:1:6.03:4.75 for mDRP-1. Antioxidant assays demonstrated that mDRP exhibited strong scavenging activity against free radicals, whereas mDRP-1 displayed weaker antioxidant capacity. Furthermore, mDRP exhibited potent inhibitory effects on α-glucosidase and α-amylase, whereas mDRP-1 showed negligible hypoglycemic activity. This discrepancy may be related to the combined influence of molecular weight, monosaccharide composition, chemical structure, surface area, and polysaccharide purification methods, though the precise mechanism requires further investigation. In summary, the in vitro data suggest that mDRP possesses significant antioxidant and hypoglycemic properties, providing preliminary evidence for further exploration and extraction of bioactive compounds. However, as these findings are derived exclusively from cell-free assays, their physiological relevance and therapeutic applicability require validation through cell-based experiments and in vivo studies before any practical applications can be proposed. Future research could focus on structural characterisation of mDRP and mDRP-1 through periodate oxidation, methylation analysis, HSQC, and COSY NMR spectroscopy, as well as investigating additional biological activities such as anti-inflammatory, antimicrobial, and antitumour effects.

## Figures and Tables

**Table 1:** Chemical composition and molecular weight of mDR and mDRP-1.

Sample	Sugar Content (%)	Protein Content (%)	Molecular Weight (Da)	Sugar (Mole Ratio)					
				Man	Rha	GalA	Glu	Gal	Ara
mDRP	75.71 ± 0.53	1.63 ± 0.28	11,811	0.43	0.27	2.58	7.6	1	1.82
mDRP-1	85.35 ± 0.27	0.36 ± 0.17	7548	0.35	0.21	0.31	1	6.03	4.75

## Data Availability

All data generated or analysed during this study were included in this article.
